# The Appropriate Combination of Hemagglutinin and Neuraminidase Prompts the Predominant H5N6 Highly Pathogenic Avian Influenza Virus in Birds

**DOI:** 10.3389/fmicb.2018.01088

**Published:** 2018-05-29

**Authors:** Xiuhui Wang, Zhaoyong Zeng, Zaoyue Zhang, Yi Zheng, Bo Li, Guanming Su, Huanan Li, Lihong Huang, Wenbao Qi, Ming Liao

**Affiliations:** ^1^National and Regional Joint Engineering Laboratory for Medicament of Zoonoses Prevention and Control, College of Veterinary Medicine, South China Agricultural University, Guangzhou, China; ^2^Department of Biomedical Sciences, College of Veterinary Medicine and Life Sciences, City University of Hong Kong, Kowloon, Hong Kong; ^3^Key Laboratory of Zoonosis, Ministry of Agriculture, College of Veterinary Medicine, South China Agricultural University, Guangzhou, China; ^4^Key Laboratory of Zoonosis Prevention and Control of Guangdong Province, Guangzhou, China

**Keywords:** H5N6, highly pathogenic avian influenza virus, appropriate combination, Major/H5, short stalk N6, predominant strains

## Abstract

Haemagglutinin (HA) and neuraminidase (NA) are two vital surface glycoproteins of influenza virus. The HA of H5N6 highly pathogenic avian influenza virus is divided into Major/H5 and Minor/H5, and its NA consists of short stalk NA and full-length stalk NA. The strain combined with Major/H5 and short stalk NA account for 76.8% of all strains, and the proportion was 23.0% matched by Minor/H5 and full-length stalk NA. Our objective was to investigate the influence of HA–NA matching on the biological characteristics and the effects of the epidemic trend of H5N6 on mice and chickens. Four different strains combined with two HAs and two NAs of the represented H5N6 viruses with the fixed six internal segments were rescued and analyzed. Plaque formation, NA activity of infectious particles, and virus growth curve assays, as well as a saliva acid receptor experiment, with mice and chickens were performed. We found that all the strains can replicate well on Madin–Darby canine kidney (MDCK) cells and chicken embryo fibroblasts (CEF) cells, simultaneously, mice and infection group chickens were complete lethal. However, the strain combined with Major/H5 and short stalk N6 formed smaller plaque on MDCK, showed a moderate replication ability in both MDCK and CEF, and exhibited a higher survival rate among the contact group of chickens. Conversely, strains with opposite biological characters which combined with Minor/H5 and short stalk N6 seldom exist in nature. Hence, we drew the conclusion that the appropriate combination of Major/H5 and short stalk N6 occur widely in nature with appropriate biological characteristics for the proliferation and transmission, whereas other combinations of HA and NA had a low proportion and even have not yet been detected.

## Introduction

H5N6 avian influenza virus (AIV) was first isolated from mallards in North America, in 1975 (Gu et al., 2011). In China, H5N6 first emerged in 2010, and its extensive circulation among both domestic and wild birds ever since has caused significant economic losses (Wu et al., 2015). Unlike H5N2 and H5N8 (Lee et al., 2014; Sun et al., 2016), which have distributed worldwide, H5N6 seemed to be confined to China, Laos, and Vietnam (Wong et al., 2015) until H5N6 infections were also reported in Japan (Hiono et al., 2017; Takemae et al., 2017) and South Korea (Kim et al., 2017). Despite no evidence that H5N6 has been transmitted to another continent, it has been detected in migratory birds (Li et al., 2017). Moreover, according to a recent study, H5N6 has replaced H5N1 as a dominant AIV subtype in ducks in southern China (Bi et al., 2016). Thus, the viruses could spread to other countries or continents via bird migration (He et al., 2017; Li et al., 2017).

**Figure 1:**
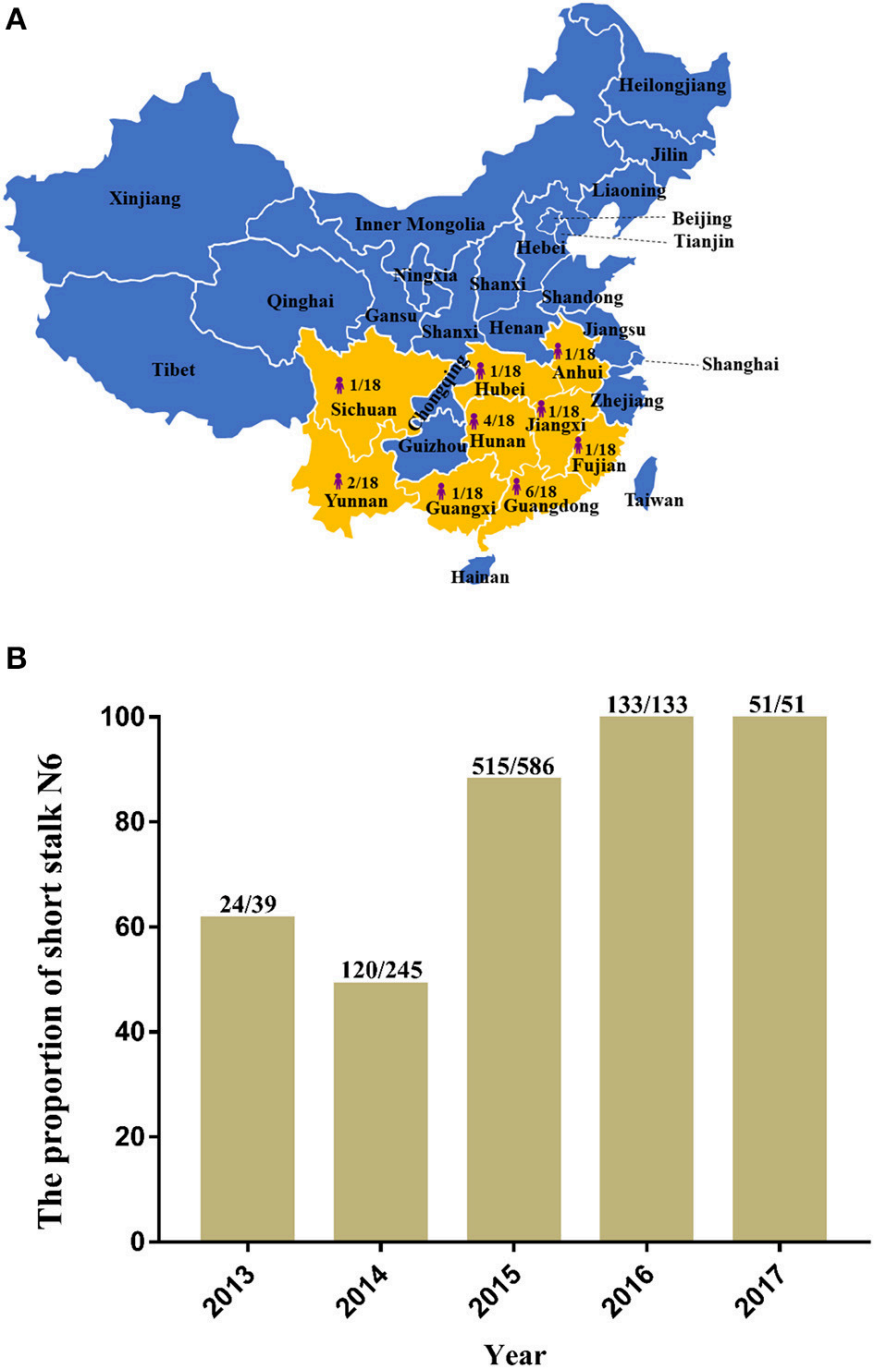
The distribution and proportion of H5N6 avian influenza viruses (AIVs) that have infected humans in mainland China and the ratio of amino acid deletion in the neuraminidase (NA) stalk of H5N6 AIVs isolated from 2013 to 2017. **(A)** Distribution and proportion of H5N6 AIVs that have infected humans in China (indicated by bright yellow on the map with the shape of a person); **(B)** Ratio of amino acid deletion in the NA stalk of H5N6 AIVs isolated from 2013 to 2017. Data were collected from January 1, 2013, to December 20, 2017, from GenBank and the Global Initiative on Sharing All Influenza Data. The numbers on the bar chart indicate the short stalk NA that accounted for the total NA in the same year.

**Figure 2:**
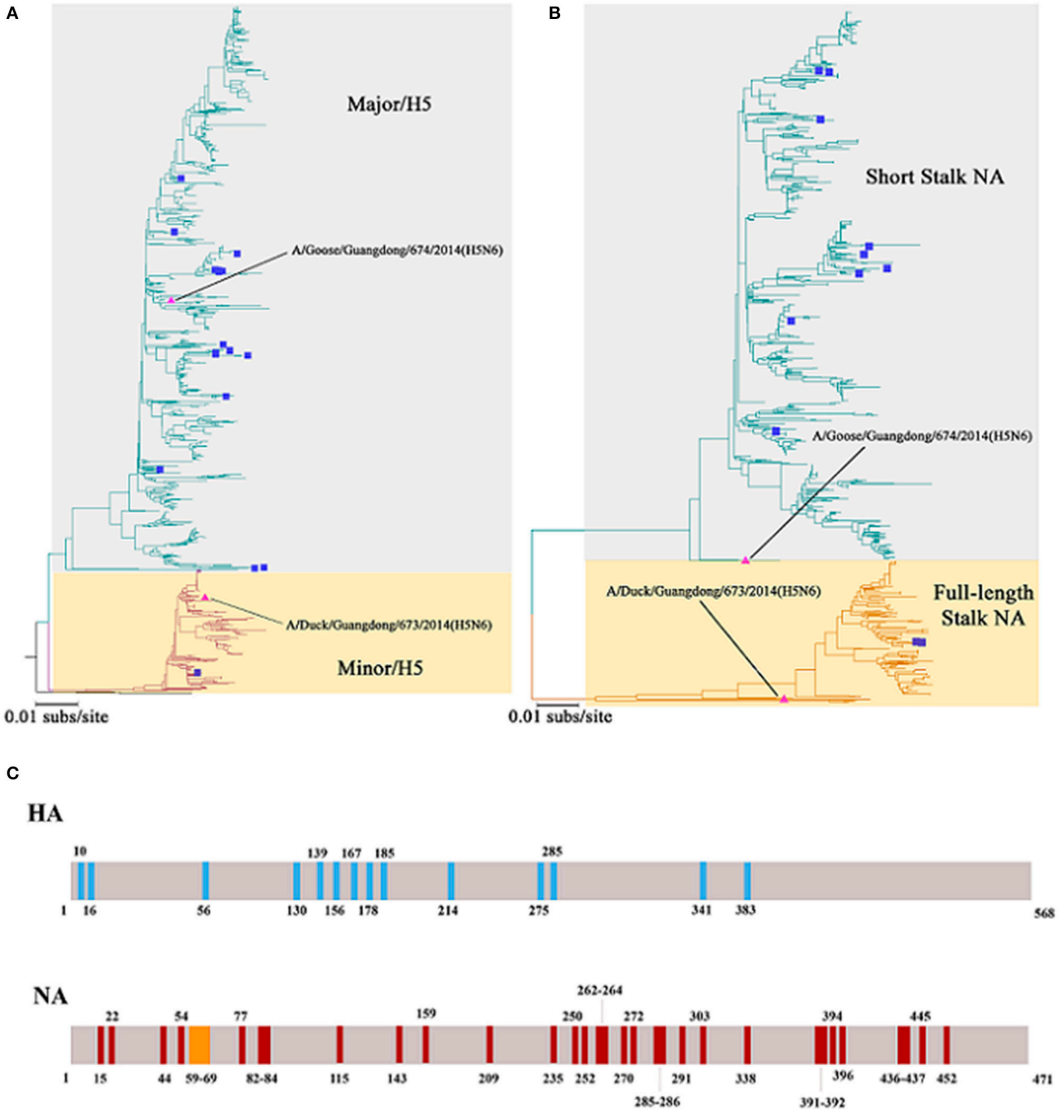
Phylogenetic trees constructed using sequences identified by the National Center for Biotechnology Information or the Global Initiative on Sharing All Influenza Data (GISAID) blast analyses and the differences of Major/Minor HA (674HA vs. 673HA) and short/full-length stalk NA (674NA vs. 673NA). **(A)** Hemagglutinin (HA) genes and **(B)** neuraminidase (NA) genes; neighbor-joining trees based on the Kimura-2 parameter model were built using MEGA (http://www.megasoftware.net/) version 6.03 with 1,000 bootstrap replicates. The scale bar represents the number of nucleotide substitutions per site (subs/site). Bootstrap values <75% are not shown in the trees. In **(A)**, the gray background represents the branches of Major/H5 and the light yellow background on behalf of Minor/H5. In **(B)**, the two colors represent short stalk NA and full-length stalk NA, respectively. The HA and NA of 673 and 674 in the study are marked by pink triangles, whereas strains that have infected humans were marked with blue squares. **(C)** The differences of Major/Minor HA (674HA vs. 673HA) and short/full-length stalk NA (674NA vs. 673NA). The colored vertical bars mean that where and how differences of Major/Minor HA (674HA vs. 673HA) and short/full-length stalk NA (674NA vs. 673NA). There are 14 amino acids difference between these two HAs at the position of 10, 16, 56, 130, 139, 156, 167, 178, 185, 214, 275, 285, 341, and 383. Compared with 673NA, 674NA contains an 11-amino acid deletion in the 59 to 69th positions of its stalk. Besides, there are 33 different amino acids between these two NAs at the position of 15, 22, 44, 54, 77, 82, 83, 84, 115, 143, 159, 209, 235, 250, 252, 262, 263, 264, 270, 272, 285, 286, 291, 303, 338, 391, 392, 394, 396, 436, 437, 445, and 452.

In recent years, H5N6 has posed a great threat to China's poultry industry. In December 2017, Hong Kong reported the detection of H5N6 infections, and more than 10,000 ducks were disposed as a result (OIE, 2017). In January 2018, a case of human infection was reported in China's Fujian Province (Fujian Provincial Health and Family Planning Commission, 2018)^1^, which raises the number of H5N6 human infections to 18 distributed throughout nine provinces in mainland China (Figure 1A), 12 of which were fatal (Lu et al., 2018). Most of the HA among the H5N6 human infection strains belong to Major/H5, and the NA belong to the short stalk NA (Figure 1B), which are features of the predominant strains of avian H5N6 (Figure 2).

HA and NA are two surface glycoproteins with vital functions when HA binds to sialyloligosaccharide viral receptors (Kaverin et al., 2002), and NA in particular removes sialic acids from host cells and viral sialyloligosaccharide (Kaverin et al., 2002). According to Bi et al., clade 2.3.4.4 of H5 includes two lineages: Major/H5 and Minor/H5 (Bi et al., 2016). Previous studies have shown that NA with special stalk motifs and HA–NA matching differ greatly among various species (Mitnaul et al., 2000; Baigent and McCauley, 2001; Li et al., 2015). Because HA and NA genes of H5N6 show apparent lineage-specific matching patterns (Bi et al., 2016), predominant strains of H5N6 warrant close attention. Given the threat of H5N6 influenza virus, understanding its pathogenic mechanism and evolution is critical.

## Materials and methods

### Virus isolation and cells

All viruses used, the H5N1 virus A/goose/Guangdong/SH7/2013 (SH7; PB2, EPI1215859; PB1, EPI1215860; PA, EPI1215861; NP, EPI1215863; M, EPI1215865; NS, EPI1215864), and the H5N6 viruses A/duck/Guangdong/673/2014 (673; HA, EPI1208375; NA, EPI980834) and A/goose/Guangdong/674/2014 (674; HA, EPI1208376; NA, EPI980835), were isolated from ostensibly healthy ducks and geese in a poultry farm in China's Guangdong Province during 2013 and 2014. Each segment used in this study is available in the Global Initiative on Sharing All Influenza Data (GISAID) database (https://www.gisaid.org/). The six internal segments of 673 and 674 were almost same and all the key amino acids which have been reported with special biological functions are identical. SH7 was used as a backbone to provide the six internal segments. Both 673HA and 674HA belong to clade 2.3.4.4 of H5N6 with 14 amino acids difference. Compared with 673NA, 674NA contains an 11-amino acid deletion in the 59th to 69th positions of its stalk (Figure 2). Therefore, strains with 674NA were marked with Δ before the virus. We inoculated the allantoic cavities of 10-day-old specific pathogen-free (SPF) chicken eggs at 37°C and froze them at −80°C after harvest. All cells used, including human embryonic kidney cells (HEK293T), Madin–Darby canine kidney (MDCK) cells, and chicken embryo fibroblasts (CEFs), were preserved by the National and Local Joint Engineering Laboratory for the Medicament of Zoonosis Prevention and Control in the College of Veterinary Medicine at South China Agricultural University (SCAU).

### Plasmid construction and reverse genetics

The two HAs and NAs of H5N6 AIVs combined with the six internal segments of SH7, respectively, and each segment was cloned into a pHW2000 plasmid system following the method of previous research (Hoffmann et al., 2000). HEK293T monolayers in 6-well plates were transfected at 80–90% confluency with 4 μg of eight plasmids (500 ng of each plasmid) by using Lipofectamine 2,000 (Invitrogen, USA) according to the manufacturer's instructions. After 4 h, the mixture was replaced with Opti-MEM (Gibco, USA) containing 0.2% bovine serum albumin. After 48 h, the supernatant was harvested and injected into SPF-embryonated eggs for virus propagation. Viruses were titrated in embryonated eggs using hemagglutination assays and sequenced.

### Phylogenetic analysis

Phylogenetic analysis was conducted with MEGA 6.0.3 using the neighbor-joining tree recommended by the World Health Organization (Liu et al., 2013). We analyzed all records of H5 and N6 uploaded by November 2017 to the GISAID and the National Center for Biotechnology Information, and a few extreme outlying sequences were removed from the trees.

### NA activity assay

NA activity of infectious particles was performed as described previously (Yu et al., 2017). Specifically, 50 μL 2-fold serial-diluted viruses were added to a 96-well flat bottom plate containing 50 μL of calcium saline buffer. No virus was added to the last well, which served as a negative control. Next, 50 μL of fluorescent substrate 2′ (4-methylumbelliferyl)-α-D-N-acetylneuraminic acid (MUNANA; Sigma) was added, and the plates were then incubated in the dark at 37°C. After 60 min, 100 μL of stop solution was added, and the results were recorded under excitation with emission wavelengths of 360 and 440 nm.

### Plaque analysis

Plaque assay was performed as previously described (Wagner et al., 2000; Tian et al., 2012; Yu et al., 2017). In particular, MDCK cells grown to 90–95% confluence in 12-well Nest tissue culture plates (Nest Scientific USA Inc.) were inoculated with a 10^−4^ dilution of virus for 2 h at 37°C; the viruses were removed and the cells washed twice in phosphate-buffered saline (PBS). And MEM containing 1% agarose (Sigma, Oakville, ON, Canada) was applied as an overlay. After 48–72 h, the cells were fixed for 5 min with 0.5% crystal violet.

### Virus growth kinetics

Virus growth kinetics of CEF and MDCK cells were gauged according to a method adapted from previous research (Xiao et al., 2016; Yu et al., 2017). Specifically, 90% confluent CEF or MDCK cells were infected at a multiplicity of infection (MOI) of 0.001, washed twice with PBS after 1 h of incubation, and incubated with 1 mL of Dulbecco's modified Eagle's medium containing 0.2% bovine serum albumin at 37°C with 5% CO_2_. Culture supernatants were collected at 12, 24, 36, and 48 h post-inoculation (h.p.i.). Viral titers were determined by the 50% tissue culture infective dose (TCID_50_) assay in MDCK cells. Statistical differences between the viruses that replaced NA compared with their respective viruses that did not (r673HA−673NA vs. Δr673HA−674NA and Δr674HA−674NA vs. r674HA−673NA) were labeled.

### Receptor-binding analysis

Receptor-binding analysis was performed as described previously by Imai et al. (Imai et al., 2012). In particular, biotin-labeled α-2,3 or α-2,6 bonds (Neu5Aca2-3LacNAcb-pAP and Neu5Aca2-6LacNAcb-pAP) were 2-fold diluted with PBS from an initial concentration of 10.00 μg/mL to a final concentration of 0.78 μg/mL. Next, 100 μL of streptavidin packaged in the labeled enzyme-linked immunosorbent assay plate according to the concentration dilution or PBS as the negative control was incubated at 4°C overnight. The unbound virus was removed, and blocked was performed by adding 200 μL 2% of skim milk–PBS at 4°C. After 8 h, 50 μL of the diluted virus with 2^6^ HA units was added to each well and incubated at 4°C, after which 100 μL of antibody against the influenza virus NP protein with 2^8^ HI was added to each well and incubated for 2 h in 4°C. Subsequently, 100 μL of diluted horseradish peroxidase-conjugated goat-anti-mouse IgG antibody was added to each well and incubated for 2 h at 4°C, after which 100 μL of tetramethylbenzidine chromogenic substrate was added, as was 50 μL 2 M sulfuric acid after 15 min to stop the reaction. The receptor specificity of HA to sialylglycopolymers was determined by measuring the absorbance at 450 nm. Each sample was measured in triplicate.

### Animal experiments

All animals were from units with production licenses, and the numbers and groupings conformed to the principles of the three Rs: reduction, replacement, and refinement. To determine the median mouse lethal dose (MLD_50_), five 4–6-week-old female BALB/c mice (Vital River Laboratories, Beijing, China) were lightly anesthetized with CO_2_ and inoculated via intranasal infection with 50 μL of 10-fold virus serial dilutions from 10^5^ to 10^1^ of 50% egg infectious doses (EID_50_). MLD_50_ values were calculated using the Reed and Muench method.

Groups of eight mice were anesthetized with CO_2_ and inoculated intranasally with 10^6^ EID_50_/50 μL of the four reassortant H5N6 viruses. Five mice in each group were monitored for body weight and clinical symptoms daily for 14 days, whereas the other three were euthanized on 4 days post-inoculation (d.p.i.). The mice were euthanized if they lost more than 25% of their initial body weight. Lung, brain, and nasal turbinate tissues of the dissected ones were harvested on Day 1 and titrated for the presence of virus by EID_50_ assay. Virus titers were compared between the full-length and short stalk NA—that is, r673HA−673NA vs. Δr673HA−674NA and r674HA−673NA vs. Δr674HA−674NA.

To understand the pathogenicity of the recombinant virus and its ability to be transmitted to poultry, 6-week-old SPF chickens were divided into four groups, each with 14 chickens. Seven chickens were intranasally inoculated with 10^5^EID_50_/200 μL of the indicated virus, whereas the other half were placed in the same cage as the contact group a day after incubation. Nine chickens inoculated with 200 μL of PBS served as negative controls. Observation of clinical symptoms was performed for all chickens for 14 days. To evaluate the difference in acutely died chickens, the first three dead chickens in each group were dissected to test the virus replication in the heart, lung, kidney, brain, spleen, and liver. Cloacal and throat swabs were collected on 2, 4, and 6 d.p.i. from both infected and contact chickens. After 14 days, serum antibody was detected for the surviving chickens.

## Facility and ethics statement

All experiments with live H5 subtype AIVs were conducted in animal biosafety level 3 laboratories and animal facilities at SCAU. All animals involved in experiments were reviewed and approved by the Institutional Animal Care and Use Committee at SCAU and treated in accordance with the guidelines of animal welfare of the World Organisation for Animal Health.

## Results

### Rescue of recombinant viruses

By using a reverse genetic system previously described (Li et al., 2012; Xiao et al., 2016; Yu et al., 2017), we rescued four H5N6 viruses. Hemagglutination assays indicated that all the rescued viruses were positive and the sequences were consistent with the original ones by sequencing. The four H5N6 viruses were named r673HA−673NA, Δr673HA−674NA, r674HA−673NA, and Δr674HA−674NA.

### Phylogenetic analysis

Phylogenetic trees revealed that 673HA and 674HA are clustered in clade 2.3.4.4 of H5N6 (Figure 2). In our study, 674HA was clustered into Major/H5 and 673HA to Minor/H5 (Figure 2A). The two NA genes formed two phylogenetic groups: short stalk NA (674NA) and full-length stalk NA (673NA) (Figure 2B). The strains of Major/H5, to which 674HA belongs, that match with short stalk NA, to which 674NA belongs, all of which we named 674-like, have become dominant strains that account for 76.8% (864 of 1,125) of all strains. Conversely, strains of Minor/H5, to which 673HA belongs, that match with full-length stalk NA, to which 673NA belongs, all of which we named 673-like, represent 23.0% (259 of 1,125) of the clade 2.3.4.4 of H5N6. On the contrary, Δr673HA−674NA-like rarely occurs in nature (Bi et al., 2016). Among the 18 human infection strains, only one belongs to Minor/H5–full-length stalk NA (Bi et al., 2016).

### NA activity assay and plaque formation assay

**Figure 3:**
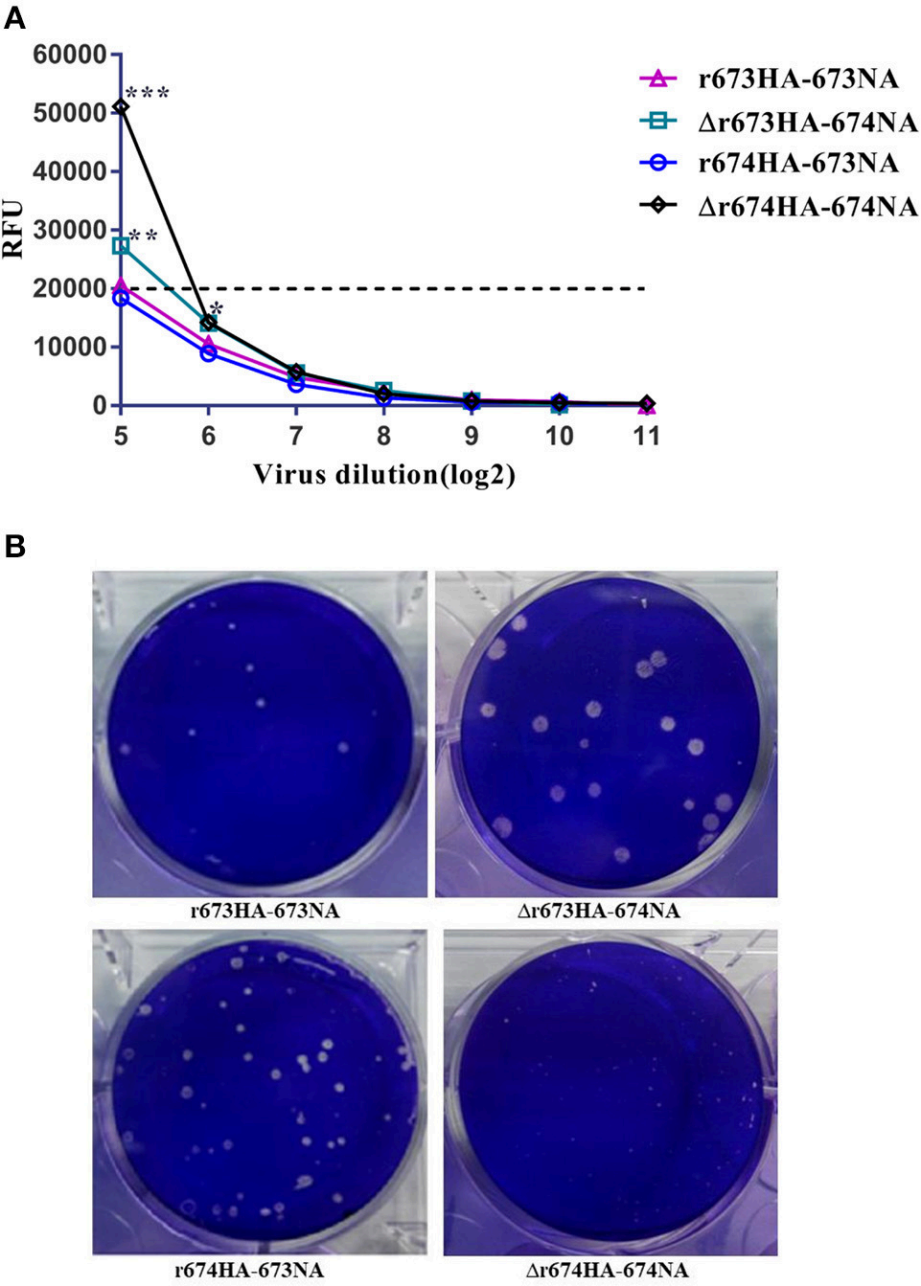
Neuraminidase (NA) activity assay and plaque analysis. **(A)** In NA activity assay of infectious particles, 50 μL of 200 μmol MUN 4-methylumbelliferone sodium salt in working concentration was added to each well after the viruses' 2-fold dilutions. The samples were placed in the dark at 37°C for 60 min, after which 100 μL of termination solution was added, and results were recorded with a microplate reader after 60 min. The dotted line represents the baseline. Statistical significance was analyzed using an unpaired *t*-test; statistical differences between the original strains and the strains that replaced NA with fixed hemagglutinin (HA)—that is, r673HA−673NA vs. Δr673HA−674NA and Δr674HA−674NA vs. r674HA−673NA were labeled (^*^p < 0.05, ^**^p < 0.01, ^***^p < 0.001). **(B)** Plaque morphology of the recombinant viruses in Madin–Darby canine kidney (MDCK) cells, whose monolayers were infected with H5N6 reassortant viruses with a 10^−4^ dilution of the virus stock for 2 h at 37°C. The monolayers were covered with an agarose-containing overlay for 48–72 h and stained with crystal violet.

Though we could not deny the possibility that noninfectious particles and free NA were present in the sample, we measured the NA activity of infectious particles. The results revealed that 674NA activity was greater than that of 673NA, regardless of its matching with 673HA or 674HA (Figure 3A). To the high pathogenic influenza virus, H5 can be automatically cleaved without trypsin (Stieneke-Gröber et al., 1992; Munster et al., 2010; Suguitan et al., 2012; Abdelwhab el et al., 2016). The results of a plaque formation assay showed that all recombinant viruses can form plaques in MDCK cells without TPCK trypsin. And plaque size differed greatly according to different strains (Figure 3B). Δr673HA−674NA grew the largest plaques (i.e., 4–5 mm), whereas Δr674HA−674NA formed pinpointed plaques in MDCK cells. The plaque size of the other two viruses was between those of Δr674HA−674NA and Δr673HA−674NA. Such results indicate that the matching of 673HA and 674NA can form large plaque.

### Growth kinetics and receptor-binding analysis

**Figure 4:**
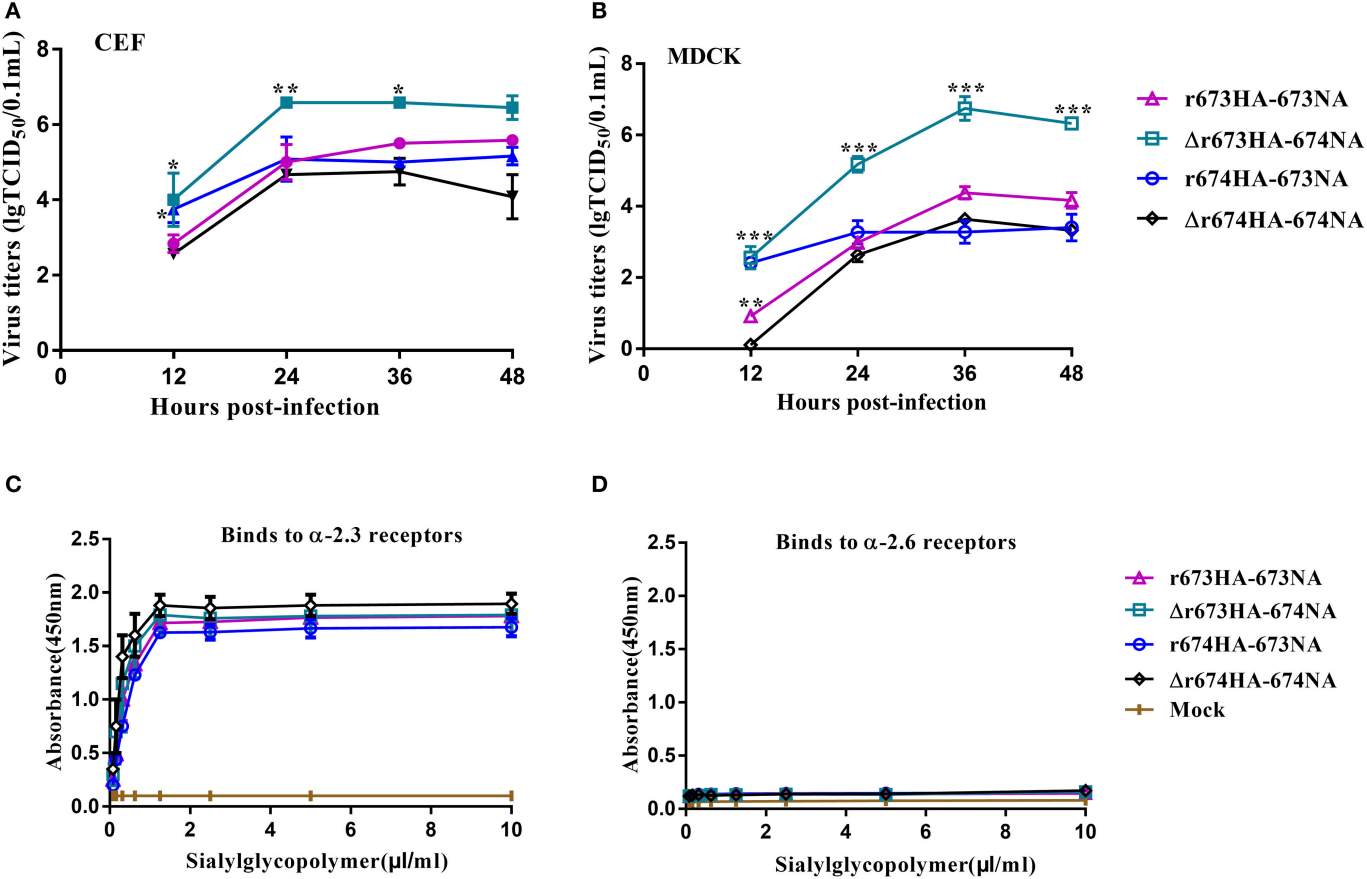
Growth kinetics and receptor-binding analysis of H5N6 reassortant viruses. Confluent **(A)** chicken embryo fibroblasts and **(B)** Madin–Darby canine kidney (MDCK) cells were infected with the indicated viruses at a multiplicity of infection (MOI) of 0.001. After 1 h incubation at 37°C, the plates were washed twice with PBS and incubated with Dulbecco's modified Eagle's medium at 39 or 37°C. The culture supernatants were harvested at 12, 24, 36, and 48 h post-inoculation (h.p.i.). Virus titers were determined by performing 50% tissue culture infective dose (TCID_50_) assays in MDCK cells. The virus titers are reported as *M* ± *SD* (*n* = 3). Statistical significance was analyzed using an unpaired *t*-test. Statistical difference between the original strains and the strains that replaced neuraminidase (NA) with fixed hemagglutinin (HA)—that is, r673HA−673NA vs. Δr673HA−674NA and Δr674HA−674NA vs. r674HA−673NA—were labeled (^*^p < 0.05, ^**^p < 0.01, ^***^p < 0.001). Binding properties to **(C)** α-2,3-glycans or **(D)** α-2,6-glycans were tested. In detail, biotin labeled α-2,3 or α-2,6 bonds were diluted 2-fold from 10.00 to 0.78 μg/mL with PBS. 100 μL streptavidin was packaged the labeled ELISA plate or PBS as the negative control, incubated at 4°C overnight. Blocked was performed by adding 200 μL of 2% skim milk–PBS in 4°C after washing 3 times with PBS. After 8 h, 50 μL of diluted virus with 2^6^ HA units was added to each well and incubated at 4°C. 100 μL of antibody against the influenza virus NP fragment with 2^8^ HI was added and incubated for 2 h at 4°C. Afterward, add 100 μL dilute Horseradish peroxidase (HRP)-conjugated goat-anti-mouse IgG antibody to each well and incubation for 2 h in 4°C, 100 μL Tetramethylbenzidine chromogenic substrate was added and 50 μL 2 M sulfuric acid was added to stop reaction after 15 min. The receptor-specificity of HA to sialylglycopolymers was determined by measuring the absorbance at 450 nm. Each sample was measured in triplicate.

To investigate the growth kinetics of the viruses, viral replication kinetics in CEF and MDCK cells were determined at 37°C with an MOI of 0.001 (Figures 4A,B). All of the viruses replicated efficiently in both CEF and MDCK cells, although especially in CEF. Δr673HA−674NA showed the highest titer in both CEF and MDCK cells, whereas the titers of r673HA−673NA and Δr674HA−674NA were moderate in both cells. Nearly all of the viruses reached their replication peak at 24 h in CEF, whereas in MDCK cells, they peaked at 36 h. Such results suggest that the viruses, especially Δr673HA−674NA, had a strong adaptability to both CEF and MDCK cells.

Receptor specificity analysis was performed to identify the affinities of the HA proteins for α-2,3-glycans and α-2,6-glycans as described previously by Imai et al. (Imai et al., 2012). These four viruses had high affinities for α-2,3-glycans, which indicates that the strains had strong tropism to avian-type receptors (Figure 4C). However, the affinity of these four viruses for α-2,6-glycans was far lower and barely caused binding (Figure 4D). Those results reveal that these four viruses have a high affinity for avian-type receptors but not human-type receptors.

### Pathogenicity of H5N6 reassortant viruses in mice

**Figure 5:**
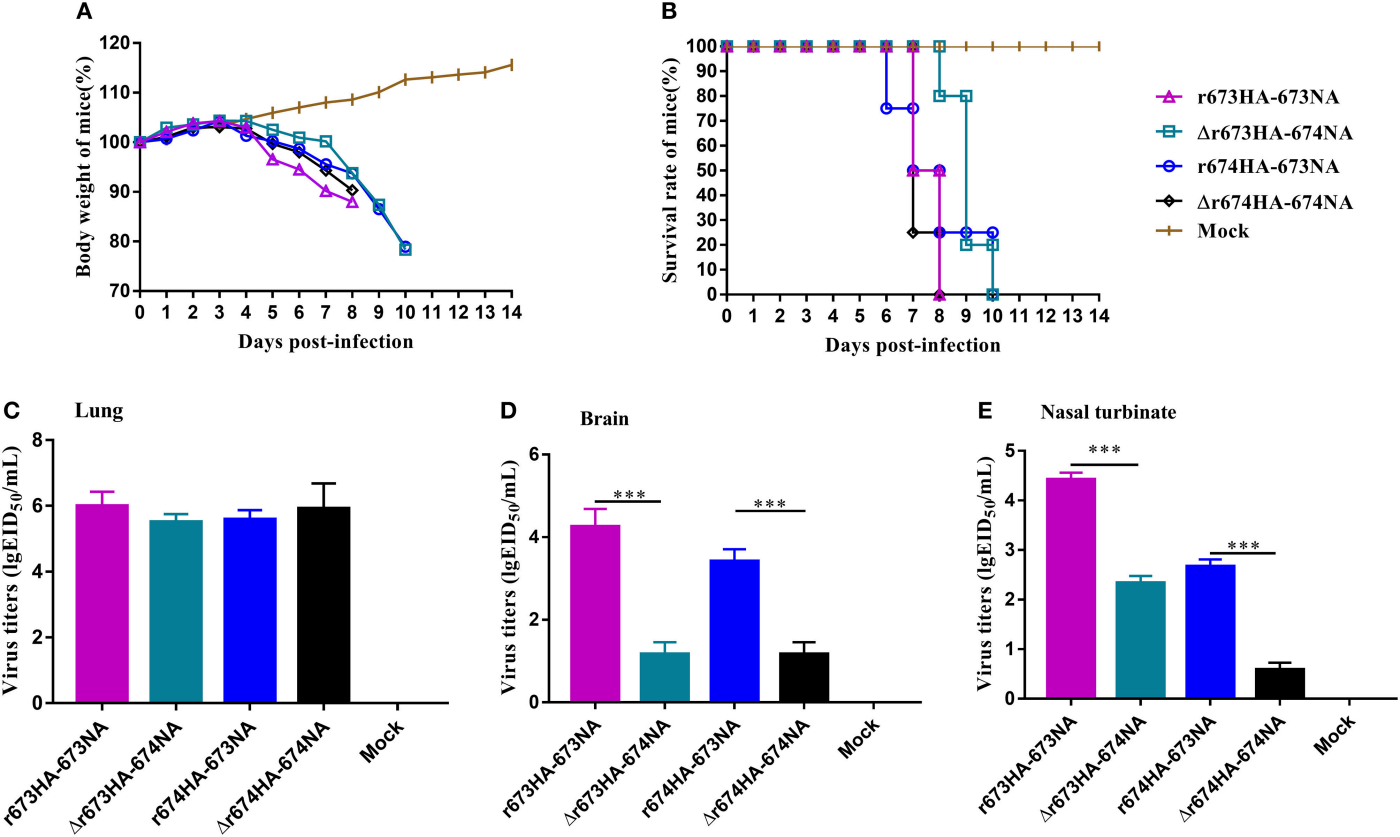
H5N6 reassortant viruses in mice. Groups of eight 4–6-week-old female BALB/c mice were intranasally inoculated with 10^6^ EID_50_/50 μL of the indicated viruses or with phosphate-buffered saline as a negative control. **(A)** Mouse body weight **(B)** and survival were monitored daily for 14 days. Mice that lost more than 25% of their initial weight were euthanized. The titers of viruses were detected in **(C)** the lung, **(D)** brain, and **(E)** nasal turbinate. To gauge the differences in strains between the full-length and short stalk neuraminidase (NA), hemagglutinin (HA) was fixed, and virus titers in tissues were determined using EID_50_ assays. Each data point represents the virus titer from an individual animal. Statistical significance was analyzed using an unpaired *t*-test, and r673HA−673NA vs. Δr673HA−674NA and Δr674HA−674NA vs. r674HA−673NA were labeled (^***^p < 0.001).

Mice were chosen as mammalian models to investigate the virulence and pathogenic of H5N6 AIVs. The MLD_50_ (log_10_EID_50/_200 μL) values of r673HA−673NA, Δr673HA−674NA, r674HA−673NA, and Δr674HA−674NA were 2.83, 4.5, 4.5, and 3.2, respectively. Groups of mice were intranasally infected with 10^6^ EID_50_/50 μL of each virus or mock infected with PBS. Weight loss occurred rapidly following infection (Figure 5A), all specimens died within 10 days (Figure 5B), and the virus could be detected in the lung (Figure 5C), brain (Figure 5D), and nasal turbinate (Figure 5E).

Little difference surfaced among the strains, and all had a higher virus titer in the lung than in other organs, which indicates that H5N6 has a high adaptability for the respiratory system. However, considerable difference did surface among the brain and nasal turbinate. In all three organs, r673HA−673NA had the highest virus titer, meaning that it can replicate well in mice. Viruses with short stalk NA (i.e., Δr673HA−674NA and Δr674HA−674NA) had lower titers in both the brain and nasal turbinate than the strains with full-length stalk NA. Those results suggest that the four H5N6 viruses were fatal to mice and had a high adaptability to the respiratory system of mice and a certain adaptability to the central nervous system. At the same time, the replication of H5N6 was influenced by both HA and NA, which suggests that HA–NA matching play an important role in the biological characteristics of influenza viruses.

### Pathogenicity and transmission of H5N6 AIVs in chickens

**Figure 6:**
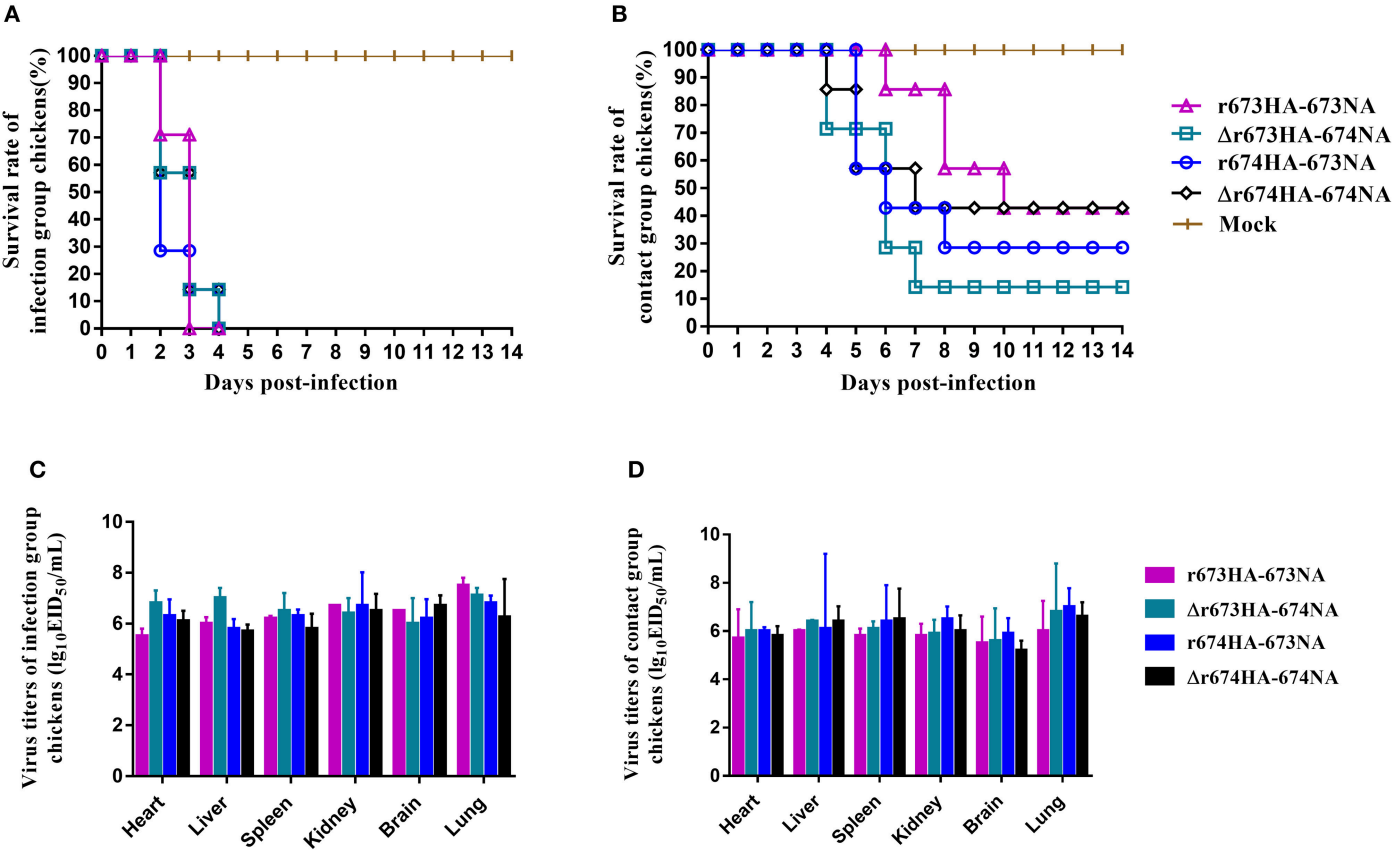
H5N6 reassortant viruses in chickens. Groups of seven 6-week-old specific pathogen-free chickens were inoculated intranasally with 10^5^ EID_50_/200 μL of the indicated viruses. A day later, seven chickens were placed in the same cage as the contact group, and nine chickens were used as negative controls. **(A)** The survival rate of chickens infected with H5N6 reassortant viruses and **(B)** the survival rate of contact chickens with the parallel infected group were determined. All chickens were observed for clinical symptoms for 14 days. Virus titers in the heart, liver, spleen, lung, kidney, and brain were tested. **(C)** Infection groups of the first three dead chickens were used for testing. **(D)** Contact groups of the first three dead chickens or of the euthanized chickens on 4 d.p.i. were observed.

The pathogenicity of the H5N6 viruses and their ability to be transmitted were also investigated in chickens. All four H5N6 strains were fatal to SPF chickens, and all chickens in infected groups died within 4 days (Figure 6A). However, differences existed among the contact groups (Figure 6B). Within a week, six chickens of Δr673HA−674NA contact group died, whereas fewer deaths occurred in the other three groups during the same period. The survival rates of contact groups infected with Δr673HA−674NA, r674HA−673NA, r673HA−673NA, and Δr674HA−674NA were 1/7, 2/7, 3/7, and 3/7, respectively. Interestingly, all chickens died within 3 days in the group infected with r673HA−673NA, but death occurred in the 6th day in the contact group, and the virus titers of throat and nasal turbinate were quite low at 2 d.p.i. Both the infected and contact groups representing Δr673HA−674NA showed a high virus titer in the throat and cloacal swabs (Table 1). All four viruses could replicate well in the organs tested whether in the infected (Figure 6C) or contact groups (Figure 6D), which indicates that H5N6 has a high tropism to chickens. The results of seroconversion were negative and revealed that the surviving chickens were not infected with the virus.

**Table 1:** Virus titers in chicken throat and cloacal swabs.

		**Virus titers (Log**_**10**_**EID**_**50**_**/200** **μL)**^**^a^**^							
		**r673HA-673NA**		**Δr673HA-674NA**		**r674HA-673NA**		**Δr674HA-674NA**	
		**Infection group**	**Contact group**	**Infection group**	**Contact group**	**Infection group**	**Contact group**	**Infection group**	**Contact group**
Throat swab	2 d.p.i.	5/5(2.0±0.4)	0/7(0)	4/4(2.3±0.8)	0/7(0)	1/2(2.5)	0/7(0)	4/4(2.1±0.5)	0/7(0)
	4 d.p.i.	—^b^	3/7(1.8±0.7)	—	3/7(2.3±0.8)	—	3/5(2.5±0.3)	—	3/6(1.5±0.1)
	6 d.p.i.	—	2/7(1.5,2.0)	—	1/3(1.5)	—	1/2(2.5)	—	1/4(1.8)
Cloacal swab	2 d.p.i.	5/5(1.7±0.3)	0/7(0)	2/4(1.5,1.7)	1/7(1.0)	1/2(1.5)	0/7(0)	3/4(1.3±0.8)	0/7(0)
	4 d.p.i.	—	2/7(1.0,1.3)	—	3/7(1.8±0.2)	—	3/5(1.8±0.5)	—	0/4(0)
	6 d.p.i.	—	2/7(1.8,1.5)	—	0/3(0)	—	0/2(0)	—	0/4(0)

*Seven 6-week-old specific pathogen-free chickens were inoculated with 10^5^ EID_50_/200 μL of H5N6 viruses. Throat and cloacal swabs were collected on 2, 4, and 6 d.p.i. from infected and naive chickens*.

a *The virus titer unit of the four strains of both throat swabs and cloacal swabs*.

b *Chickens have died out at sample collecting time point*.

## Discussion

According to phylogenetic analysis, the HA genes of 673 and 674 are clustered in clade 2.3.4.4 of H5N6 yet belong to Minor/H5 and Major/H5, respectively. The two NA genes form two phylogenetic groups: one with 11 amino acid deletion in the NA stalk (i.e., 674NA), the other with a full-length stalk (i.e., 673NA). Further analysis showed that the strains combined with Major/H5 and short stalk N6 (i.e., 674-like) have become the dominant ones. The strains with Minor/H5 and short stalk N6, by contrast, rarely occur in nature (Bi et al., 2016), possibly because the virus with Minor/H5–short stalk NA matching has high pathogenicity, and consequently, hosts were killed before the virus completely adapted to them. In any case, its likelihood of infecting hosts is less. Therefore, although Major/H5–short stalk NA matching is relatively less pathogenic, the strains can coexist with hosts, which increases the possibility of infection. That phenomenon can be affected by two parts: First, the evolution of HA and NA genes. As HA and NA may be affected by the internal segments (Lindstrom et al., 1998). Second, fewer descendants might generate by some reassortment events (Villa and Lässig, 2017).

A series of studies *in vitro* were performed. We found that Δr673HA−674NA formed the greatest plaque on MDCK cells and replicated well on both CEF and MDCK cells. While the plaque size of Δr674HA−674NA was smaller than that of Δr673HA−674NA and virus titers in MDCK and CEF much lower. And these features of r673HA−673NA were between Δr673HA−674NA and Δr674HA−674NA.Those findings illustrate that HA–NA matching plays an important role in cultured cells.

All BALB/c mice and SPF chickens in the infected groups died during the period of observation, thus indicating that those H5N6 strains can be fatal to both mammals and poultry. The viruses demonstrate strong tissue tropism to both the lung and nasal turbinate of mice, although the former tropism is far higher than that of the latter. At the same time, H5N6 can also intrude into the central nervous system and be detected in the brain. Moreover, strains with full-length stalk NA have higher virus titers in the brain of mice than strains with short stalk NA. The same phenomena also occurred in nasal turbinate. In chicken assays, Δr673HA−674NA showed not only high pathogenicity to chickens but also a high virus titer in swabs (Table 1) and organs (Figure 6), which suggests that the strain has strong virulence. However, according to phylogenetic analysis, the strain has not yet been found in nature. Chickens infected by Δr673HA−674NA died quickly, which could have been detrimental to the proliferation and effective transmission of the virus. Conversely, due to the high survival rate of r673HA−673NA and Δr674HA−674NA, more chickens were survived. Therefore, the viruses can be transmitted by the surviving chickens from one place to another, and the viruses are conducive to transmission and can spread via surviving chickens.

Previous studies have revealed that the balance of HA binding and NA cleavage bears an important influence on the replication of tissue culture (Baigent and McCauley, 2001), embryonated chicken eggs (Mitnaul et al., 2000), transmission in chickens (Yu et al., 2017) and ferrets (Yen et al., 2011), and pathogenicity in mice (Gen et al., 2013), as commonly seen in H1N1 (Yen et al., 2011), H3N2 (Richard et al., 2012), and some other subtypes such as H5 and H7 (Baigent and McCauley, 2001). We found that HA–NA matching can promote H5N6 replication in tissues and transmission in chickens. In our study, phylogenetic analysis revealed that the four combination branch strains differ greatly in proportion. The results in mice were inconsistent with those of tissue cultures and in chickens, perhaps because all viruses in our study were from birds and currently unable to adapt to mammals, as suggested by Casalegno's findings as well (Casalegno et al., 2014).

NA with stalk deletion occurs widely in H1N1 and H5N1, and the special NA stalk motif is responsible for increasing the virulence and pathogenesis of these two subtypes (Baigent and McCauley, 2001; Zhou et al., 2009). Short stalk N6 forms a large proportion of total N6 and shows an increasing trend, and the strains combined with Major/H5 and short stalk N6 have become predominant. To HA, though has a low affinity to α-2, 6 sialic acid receptors, under environmental pressure in the form of vaccine selection the virus is apt to achieve adaptive mutations and show a strong affinity to both α-2, 3 and α-2, 6 sialic acid receptors, which can cause considerable damage to livestock breeding and human health (Sun et al., 2016).

Altogether, the appropriate matching of HA and NA can promote H5N6 replication in tissue cultures and transmission in chickens. Though the rise of short stalk NA could also contribute to the enhancement of those properties, especially when it combines with Minor/H5, to exist for a long time, HA–NA balance is essential to maintain good viral fitness. Thus, H5N6 highly pathogenic influenza viruses need to co-operate with hosts and meet the requirements of proliferation and transmission. That finding is critical to the understanding of H5N6 evolution and prevention. Close attention should be paid to prevent any further mutation of the recombinant strains in nature and to the viruses to prevent their breaking the species barrier and consequently infecting humans.

## Author contributions

XW and ZhZ contributed equally to this work. XW, ZhZ, WQ, and ML conceived and designed experiments. XW, ZhZ, ZaZ, YZ, BL, GS, LH, HL, WQ, and ML performed the experiments. XW, ZhZ, ZaZ, WQ, and ML conducted phylogenetic and data analyses. XW, WQ, and ML prepared and revised all figures. XW, ZhZ, ZaZ, YZ, BL, GS, LH, HL, WQ, and ML wrote and modified the manuscript. All authors contributed to the study and have read and approved the final manuscript.

### Conflict of interest statement

The authors declare that the research was conducted in the absence of any commercial or financial relationships that could be construed as a potential conflict of interest.

## Footnotes

^1^Available online at: (http://www.fjhfpc.gov.cn/xxgk/gzdt/wsjsyw/201801/t20180105_713904.htm).
